# Integrating partonomic hierarchies in anatomy ontologies

**DOI:** 10.1186/1471-2105-5-184

**Published:** 2004-11-26

**Authors:** Albert Burger, Duncan Davidson, Yiya Yang, Richard Baldock

**Affiliations:** 1 MRC Human Genetics Unit, Western General Hospital, Crewe Road, Edinburgh EH4 2XU, UK

## Abstract

**Background:**

Anatomy ontologies play an increasingly important role in developing integrated bioinformatics applications. One of the primary relationships between anatomical tissues represented in such ontologies is *part-of*. As there are a number of ways to divide up the anatomical structure of an organism, each may be represented by more than one valid partonomic (part-of) hierarchy. This raises the issue of how to represent and integrate multiple such hierarchies.

**Results:**

In this paper we describe a solution that is based on our work on an anatomy ontology for mouse embryo development, which is part of the Edinburgh Mouse Atlas Project (EMAP). The paper describes the basic conceptual aspects of our approach and discusses strengths and limitations of the proposed solution. A prototype was implemented in Prolog for evaluation purposes.

**Conclusion:**

With the proposed name set approach, rather than having to standardise hierarchies, it is sufficient to agree on a suitable set of basic tissue terms and their meaning in order to facilitate the integration of multiple partonomic hierarchies.

## Background

### Introduction

As the bioinformatics emphasis has shifted from gene sequence analysis to functional genomics and proteomics, the need to describe gene function in the context of specific tissues of an organism has increased. Hence, in addition to anatomy ontologies built for medical purposes, e.g. GALEN [1], descriptions of anatomies are now often used to annotate a variety of genetic data, such as gene-expression. (A list of such ontologies for human as well as model organisms, e.g. mouse, *Drosophila*, zebrafish and *C elegans*, can be found on the Open Biological Ontologies web site [2].

An ontology model typically consists of concepts and relationships between these concepts. One of the key relationships in anatomy is *part-of*. It is possible to distinguish between different kinds of *part-of*, e.g. *structural part-of *and *functional part-of*. Each anatomy ontology may define one or more such *part-of *relationships.

Even for a single type of part-of, there may be more than one correct way to devide the anatomy of an organism into parts and subparts. Hence, multiple valid partonomic (part-of) hierarchies may exist for any organism. This raises the issue of interoperability across such hierarchies: when is a tissue in one hierarchy *equivalent *to a tissue in another hierarchy, and what are the *part-of *relationships across these hierarchies?

In general, biologists refer to tissues by their names, unlike computers which can easily work with ID numbers. For example the name "/embryo/limb/forelimb bud/ectoderm" is used to describe the ectoderm of the forelimb bud of the limb of the mouse embryo. Although this name uniquely identifies the tissue, it does so by encoding the particular partonomic hierarchy in the name. This causes problems when trying to work with more than one single hierarchy. This paper discusses a naming scheme that preserves the unique identification property of tissue names, without having to restrict it to a particular hierarchy, thus making it easier to integrate multiple partonomic hierarchies.

There is a large body of work discussing mereology (part-of relationships) in the biomedical literature. For example, Rogers and Rector [3] describe their experience of modelling part-of relationships in human anatomy as part of the GALEN project. Aspects of the Digital Anatomist Foundational Model (FMA) are given in [4]. Partonomies in a 3D model of human anatomy are briefly discussed in [5]. All of these papers distinguish between different kinds of part-of relationships. An example of an anatomy ontology using only one type of part-of can be found in the Edinburgh Mouse Atlas Project (EMAP). Although EMAP also uses derives-from relationships to capture cell lineage information with respect to embryo development, it is significantly less complex than GALEN and the FMA. Such variation of complexity is common and typically reflects the different purposes for which the ontologies were built. The EMAP ontology is used to label spatial data for the developing mouse embryo, specifically gene expression data [6].

We are not aware of any previous work dealing specifically with the integration of multiple part-of anatomy hierarchies. However, ontology alignment and integration in general is an active reserach area and has produced tools that aim at helping with the manual alignment of ontologies as well as with the automation of ontology integration. Examples of such tools include OntoMorph [7], OntoMerge [8] and the PROMPT tools suite [9]. Some work has been carried out in trying to use such tools to systematically merge GALEN and FMA, but the results have been rather limited [10,11]. In this paper we are not trying to argue for a general solution to the ontology integration problem, which as the evidence suggests is very hard to achieve. Instead we approach the problem from our specific application experience and seek to find a specific solution for a more limited domain.

The remainder of the paper is organised as follows. The next section introduces the Edinburgh Mouse Atlas, which forms the basis of the work presented here. Thereafter, the issue of multiple part-of hierarchies is discussed. The next section introduces the developed name set representation, followed by a discussion that covers the implementation of a Prolog prototype system. The proposed approach is then evaluated in the discussion section, followed by our conclusions.

### Edinburgh Mouse Atlas

The Edinburgh Mouse Atlas (EMAP) and Gene Expression (EMAGE) Database project [12-16] has developed a digital atlas of mouse development which provides a bioinformatics framework to spatially reference biological data. The core databases contain 3D grey-level reconstructions of the mouse embryo at various stages of development, a systematic nomenclature of the embryo anatomy (the anatomy ontology), and defined 3D regions (domains) of the embryo models which map the anatomy onto the spatial models. Through the 3D domains users can navigate from the spatial representation of the embryo to the ontology and vice versa. Data from an *in situ *gene expression database is spatially mapped onto the atlas allowing the users to query gene expression patterns using the 3D embryo model and/or the ontology as a reference.

**Figure 1:**
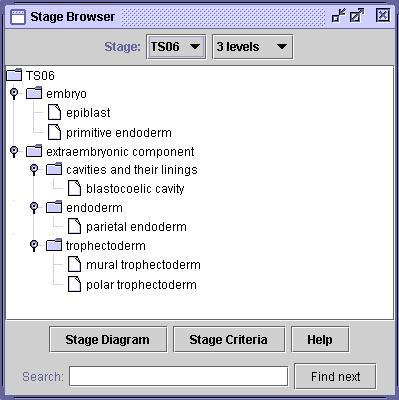
**Anatomy browser **Screenshot of Mouse Atlas anatomy browser showing the top 3 levels of mouse embryo anatomy at developmental stage TS6.

Following the description of mouse embryo development by Theiler [17], the anatomy ontology is organised into 26 developmental stages, referred to as *Theiler stages *(TS1-TS26). Each stage is primarily organised as a *structural part-of *tree, or partonomic hierarchy. Figure 1 shows the top 3 levels of the tree at TS6. (The browser shown in the figure is available on-line at the Mouse Atlas web site [12].)

The tissues represented by subnodes of a node in the tree are intended to be *non-overlapping *(*exclusive*) and *complete*, i.e. they describe *all distinct *parts of the parent tissue. For example, in Figure 1, the trophectoderm consists of the mural trophectoderm and the polar trophectoderm, which are *distinct *from each other and are the *only *parts of the trophectoderm at that stage. Although this holds for EMAP, it is not a requirement for the proposed approach. (In this paper, the term 'tissue' is used in a very generic way, meaning both: whole anatomical structures as well as specific tissues.)

Each tissue can be uniquely identified by its *full name*. A *full name *is an n-tuple: (*t*_0_, *t*_1_,...,*t*_*n*_). The *path name *of the tissue is (*t*_0_, *t*_1_,..., *t*_*n*-1_). The component name is *t*_*n*_. For example, given the tissue name (using a file directory style notation):

/embryo/branchial arch/3rd arch/branchial pouch/endoderm/dorsal

its full name is:

(embryo, branchial arch, 3rd arch, branchial pouch, endoderm, dorsal)

its path name is:

(embryo, branchial arch, 3rd arch, branchial pouch, endoderm)

and its component name is:

dorsal

**Figure 2:**
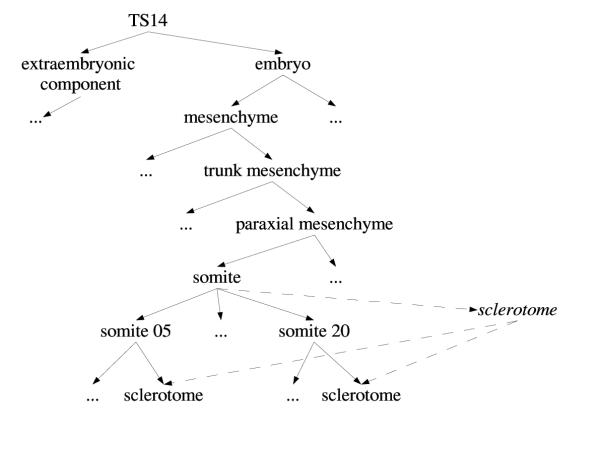
**Extract of part-of hierarchy in EMAP at Theiler Stage 14 **Diagram illustrating the need for so-called "group" nodes, sclerotome in this example, in the EMAP anatomy part-of hierarchy.

Although the ontology covers all parts of the mouse embryo, there may not be a single node representing a particular tissue of interest. For example, there is no single node named (embryo, mesenchyme, trunk mesenchyme, paraxial mesenchyme, somite, sclerotome). However, there is a tissue named (embryo, mesenchyme, trunk mesenchyme, paraxial mesenchyme, somite), which has somite 05 to somite 20 as subparts (somite 05 to somite 20 are part of that tissue), and each of those has a subpart with component name sclerotome. The approach taken in EMAP is to introduce a new tissue node, called a *group*, with the appropriate subparts identified. Figure 2 shows the anatomy part-of graph for this example (at Theiler stage 14).

Although adding the notion of *groups *to EMAP is addressing the need for alternative arrangements of the part-of hierarchy, it does also raise a number of new questions. For example, it requires a suitable algorithm to determine appropriate tissues of which the new group should be part of. Also, some restrictions should be put in place to constrain what new links can be added; for example, if a new group contains all parts of some other tissue, then that tissue itself, rather than all of its parts, should be linked to the group. In other words, we require a mechanism that prevents biologists from adding too many part-of links unnecessarily. Let us assume that a new group needs to be introduced that contains leg as one of its parts. In this case the biologist should introduce a single part-of link between the new group and leg, and not multiple part-of links between the new group and hip, knee, lower leg and upper leg (which are the parts a leg consists of). The fact that these are parts of the group should be deduced from the transitivity property of the part-of relationship. These and other considerations seem representative of the more general problem of trying to integrate multiple part-of hierarchies over the same anatomical space. The remainder of this paper describes a possible solution to this problem.

### Multiple part-of hierarchies

As previously mentioned, there is more than one way to structure the anatomical part-of hierarchy of an organism. The intersection of these hierarchies may occur at any level; they may share some or all of the their leaf nodes, but may also share intermediate nodes. A particular hierarchy may only deal with part of the organism, e.g. brain or heart, while others, such as EMAP, cover the entire organism.

The central example we use in this paper is that of somites. The somites are a repeating anatomical structure down the back of the animal. They give rise to the vertebrae, muscles of the backbone, skin and other structures. Each somite is divided into 3 parts: dermomyotome, myocoele and sclerotome. The dermomyotome is a group of cells which form the dermal layer of the skin and muscle tissue. The myocoele is a fluid-filled cavity of the somite, and the sclerotome gives rise to the bone of the vertebrae.

Most ontologies require each of their concepts to be uniquely identified by a name. In the context of an anatomical ontology, such as EMAP, it is clearly not enough to simply use the name sclerotome when wanting to refer to the sclerotome of somite 18. In general, the full name of the tissue is required, though in some cases a part of it may be sufficient, e.g. there is only one tissue at Theiler stage 14 that has component name somite 18.

**Figure 3:**
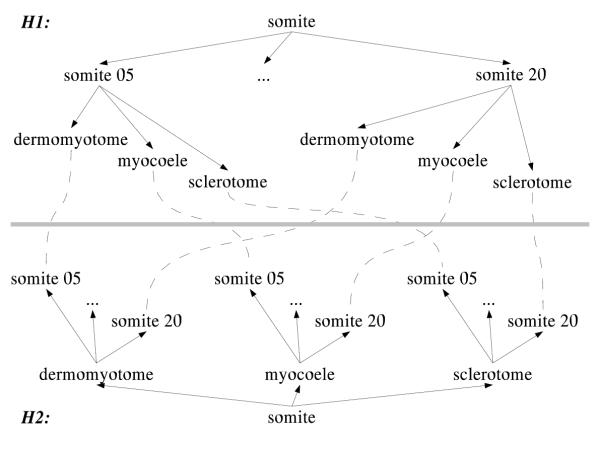
**Alternative hierarchies for somite **Two possible part-of hierarchies for the somite part of the ontology, and how they relate.

Focusing on the somite part of the anatomy given in Figure 2, we can draw two possible hierarchies, as shown in Figure 3. (somite, somite 05, dermomyotome) in H1 and (somite, dermomyotome, somite 05) in H2 clearly semantically refer to the same mouse embryo tissue, in spite of using different names. Hence, for an anatomy ontology to embody its particular part-of hierarchy in the naming of its tissues is not helpful when it comes to integrating multiple hierachies. The proposal is therefore to avoid this problem by using name sets to identify a particular tissue.

## Results

### Name set representation of part-of hierarchies

#### Basic name sets

Each tissue in a part-of hierarchy is identified by the set of component names along the path from the root to the tissue (including the component name of the tissue itself). For example, in H1 the dermomyotome of somites 5 and 20 are represented as {dermomyotome, somite, somite 05} and {dermomyotome, somite, somite 20}, respectively; and in H2 somite 20's dermomyotome is represented as {dermomyotome, somite, somite 20}. Using NS(T) to denote the name set of tissue *T*, equivalence between two tissues is identified by the equivalence of their name sets:

*NS*(*T*_*i*_) = *NS*(*T*_*j*_) → *T*_*i *_= *T*_*j*_

Let *T*_*i*_
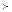
* T*_*j *_denote that *T*_*i *_has *T*_*j *_as a direct subpart, and let *T*_*i *_
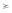
*T*_*j *_denote that *T*_*i *_has *T*_*j *_as a subpart (direct or indirect)^1^, i.e. *T*_*i *_ 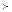
*T*_*j *_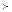... 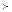
*T*_*k *_implies *T*_*i *_
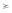
*T*_*k*_, then the part-of relationships can be derived from the name sets as follows:

*NS*(*T*_*i*_) ⊂ *NS*(*T*_*j*_) → *T*_*i *_
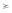
*T*_*j *_and

*T*_*i *_
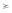
*T*_*j *_∧ (¬ ∃*k*·*T*_*i *_
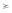
*T*_*k *_∧ *T*_*k *_
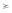
*T*_*j*_) → *T*_*i *_
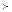
*T*_*j*_

The first line simply states that *T*_*i *_has *T*_*j *_as a subpart, if the name set of the first is a proper subset of the name set of the second. The second line states that *T*_*i *_has *T*_*j *_as a direct subpart (or child tissue) if *T*_*i *_has *T*_*j *_as one of its subparts, and there are no other subparts of *T*_*i *_which themselves have *T*_*j *_as one of their own subparts. In the graph representing the ontology, an arrow is drawn from *T*_*i *_to *T*_*j *_if, and only if, *T*_*i *_
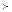
*T*_*j*_.

The name set representation does not explicitly deal with temporal relationships. For example, changes in the anatomy of the developing embryo must be captured explicitly, i.e. if a particular subpart disappears from one developmental stage to the next, this should be reflected in the lack of that subpart in the ontological representation for the latter stage. Furthermore, the given representation does not explicitly distinguish between *classes *and *instances *of tissues. For example, while in general it holds that a leg has a lower leg part, this may not be true in specific instances. The proposed representation does not deal with such instance issues; many of the existing model organism anatomy ontologies used in bioinformatics today similarly do not represent information at the instance level.

#### Rest-of tissues

**Figure 4:**
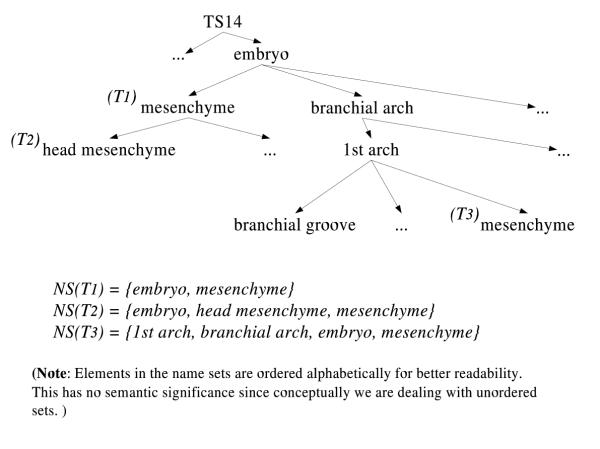
**Name set representation and part-of hierarchy **The name set representations for selected tissues are presented in the context of the part-of hierarchy.

A "rest-of" tissue is a tissue that represents all parts of that tissue other than those which are explicitly represented in a "sibling" of the rest-of tissue. For example, the embryo mesenchyme marked as *T*_1 _in Figure 4 does not include the mesenchyme of the first branchial arch (labeled *T*_3_) or any of the other parts of the embryo (not shown in the figure).

Looking at the name set representation of *T*_1 _and *T*_3 _(in Figure 4), we see that *NS*(*T*_1_) ⊂ *NS*(*T*_3_). Based on the definition from above, *T*_1 _
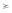
*T*_3 _follows. This, however, is not true. In other words, the basic name set representation introduced earlier is not sufficient to cope with rest-of tissues.

#### Positive and negative name sets

To deal with "exclusions" such as required for rest-of tissues, we introduce *negative name sets *(*NS*_*n*_) in addition to the name sets we already have (and we shall refere to as *positive name sets *(*NS*_*p*_) from now on). A tissue *T*_*r *_includes in its negative name set the component name of any "sibling" tissue *T*_*s*_, if *T*_*s*_has a subpart with the same component name as *T*_*r*_. For example, branchial arch is added to the negative name set of *T*_1 _because of *T*_3 _(from Figure 4).

Part-of relationships can now be derived from the name set representation of tissues as follows:

*NS*_*p*_(*T*_*i*_) ⊂ *NS*_*p*_(*T*_*j*_) ∧ *NS*_*n*_(*T*_*i*_) ∩ *NS*_*p*_(*T*_*j*_) = ∅ → *T*_*i *_
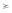
*T*_*j *_and

*T*_*i *_
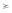
*T*_*j *_∧ (¬ ∃ *k*·*T*_*i *_
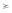
*T*_*k *_∧ *T*_*k *_
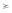
*T*_*j*_) → *T*_*i *_
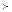
*T*_*j*_

The first line states that *T*_*i *_has *T*_*j *_as a subpart, if the positive name set of *T*_*i *_is a proper subset of the positive name set of *T*_*j*_, and the intersection of the positive name set of *T*_*j *_and the negative name set of *T*_*i *_is empty. The intersection part has been added to enforce the exclusions needed to deal with rest-of cases. The second line's meaning is identical to what it was before.

Returning to the example in Figure 4, *T*_1 _is now represented as *NS*_*p*_(*T*_1_) = {embryo, mysenchyme} and and *NS*_*n*_(*T*_1_) = {branchial arch}, *T*_3 _is represented as *NS*_*p*_(*T*_3_) = {1st arch, branchial arch, embryo, mesenchyme} and *NS*_*n*_(*T*_3_) = {}. Since *NS*_*n*_(*T*_1_) ∩ *NS*_*p*_(*T*_3_) = {branchial arch}, i.e. non-empty, *T*_3 _is not a subpart of *T*_1_, as required.

For exclusions to work properly, negative name sets must be propagated to their subparts, as is implicitly the case for positive name sets already. Hence, *T*_2 _(in Figure 4) will also include branchial arch in its negative name set, keeping *T*_3 _from becoming one of its subparts.

### Integration of multiple part-of hierarchies

**Figure 5:**
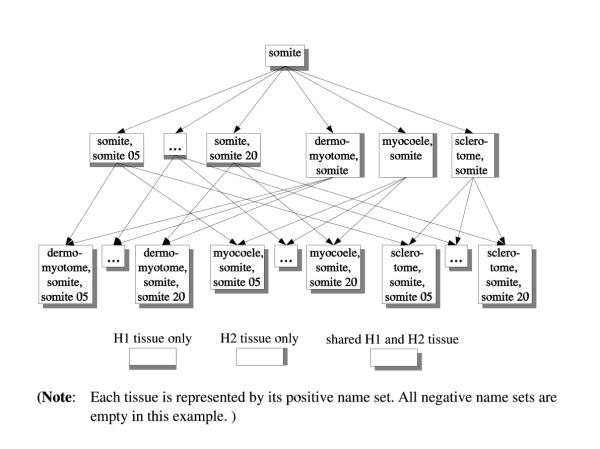
**Merged ontology for somite **The part-of hierarchy that results from merging two possible hierarchies for somites.

Assuming that two or more part-of hierarchies are based on the same set of component names, integrating these hierarchies becomes a trivial task. Relationships (identity as well as part-of) between tissues from different hierarchies follow directly from the rules described above. For example, applying these rules to the hierarchies in Figure 3, the integrated part-of hierarchy of Figure 5 can automatically be generated.

Given the integrated name set representation of two or more hierarchies, it is not possible to determine which tissue belongs to which original hierarchy. For example, if asked for the immediate subparts of somite, based on the rules governing the part-of relationship, all of the tissues at the second level of the diagram in Figure 5 would be returned. To address this problem, extra information needs to be captured. This can easily be achieved by adding a *view set *to each tissue. For example, the view set for somite would be {H1, H2}, as it would be for all leaf node tissues in Figure 5. The intermediate tissue nodes have either {H1} (left part) or {H2} (right part) as their view sets. Thus, recreating one of the original hierarchies simply becomes a matter of filtering the integrated hierarchy using the view sets. In addition to the reconstruction of the original hierarchies, new views on the integrated hierarchy, or even on the original ones, can easily be created using appropriate name set "queries".

### Prototype

A prototype of the name set representation for the Mouse Atlas anatomy ontology has been implemented in Prolog; an extension of the prototype we developed for our work on the *Formalisation of Mouse Embryo Anatomy *[6]. This original prototype included the following two predicates:

tissue(S, T, FN).

• S: stage ID, e.g. 14 for Theiler stage 14;

• T: tissue ID number (accession number);

• FN: full name of tissue represented by the list [N1, N2, N3, ...]; 

hasPart(TID1, TID2).

• TID2 is an immediate part of TID1, i.e. *TID*1 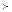
*TID*2;

For the evaluation of the name set representation, we use an extended version of the tissue predicate (view handling is ommitted from the protoype description to keep our examples simple) :

ext_tissue(S, T, FN, NSp, NSpL, NSn, NSnL).

• S, T, FN: as above;

• NSp: positive name set of tissue represented by a list [N1, N2, N3, ...];

• NSpL: length of NSp;

• NSn: negative name set of tissue represented by a list [N1, N2, N3, ...];

• NSnL: length of NSn;

For example, the embryo mesenchyme tissue of Figure 4 is represented as:

ext_tissue(14,705, ["embryo", "mesenchyme"],

["embryo", "mesenchyme"], 2,

["branchial arch", "limb", "organ system"], 3).

The following Prolog clause is used to determine whether *T*_*p *_
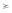
*T*_*c *_is true:

subPart(Tp, Tc) :-

ext_tissue(Sp, Tp,_, NSpp, NSpLp, NSnp,_),

ext_tissue(Sp, Tc,_, NSpc, NSpLc,_,_),

NSpLc > NSpLp,

ord_subset(NSpp, NSpc),

ord_disjoint(NSpc, NSnp).

Predicates ord_subset and ord_disjoint from the Prolog library were used to implement the set theoretic aspects of the representation. Although these predicates support ordered sets, this is not required for our representation (but there were no unordered set predicates in the library). NSpLc > NSpLp is required to enforce *proper subset *relationships.

The following two Prolog clauses are used to determine whether *T*_*p *_
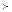
*T*_*c *_is true:

not_immediate_subPart(Tp, Tc) :-

subPart(Tp, Tm),

subPart(Tm, Tc).

immediate_subPart(Tp, Tc) :-

subPart(Tp, Tc),

not not_immediate_subPart(Tp, Tc).

The Prolog implementation given is not particularly efficient and there are a number of optimisations that could be put in place. However, as the purpose of the prototype was not to deliver a robust application for end-users, but a reference implementation of the proposed approach for evaluation purposes, it proved entirely sufficient.

The paper makes no claims over the relative merits of different implementation strategies for the proposed approach. Alternatives to Prolog include using a relational database system or an ontology language, such as OWL (more details of OWL available from W3C [18]). The latter is of particular interest as it is gaining wide acceptance in the bioinformatics domain. At the time this work began, tools for developing ontologies using OWL were still in their early stages, and hence, we decided not to use them. In the meanwhile, however, Protege [19] and OilEd [20], have matured sufficiently and do provide appropriate alternative implementation platforms.

## Discussion

For evaluation purposes, a number of tests were carried out on the name set representation of the Mouse Atlas anatomy. These are discussed here, together with some general observations about the proposed approach.

The first assumption that must hold is that no two tissues (at any given stage) have the same name set representation. This was tested using

test1 :-

ext_tissue(S, T1,_, NSp,_, NSn,_),

ext_tissue(S, T2,_, NSp,_, NSn,_),

T1 not = T2.

test1 returns no, i.e. no two different tissues with the same name sets were found, as required.

To test whether all part-of relationships can be reconstructed from the name set representation, we used

test2 :- immediate_subpart(T1, T2), not hasPart(T1, T2).

test3 :- hasPart(T1, T2), not immediate_subPart(T1, T2).

Both, test2 and test3 return no, i.e. the name set representation does not lead to any part-of relationships that are not intended (test2), and all existing part-of relationships are found through the name sets (test3), as required.

The smallest form of part-of hierarchy integration is the addition of a new tissue node, which is equivalent to adding a *group *in EMAP. A recently identified need for a group has been for all (embryo, mesenchyme, trunk mesenchyme, paraxial mesenchyme, somite, myocoele) tissues at Theiler stage 17. Using predicate

immediate_subPart_ns(S, NSp, NSn, T).

• S: stage ID;

• NSp: positive name set of new tissue node (group);

• NSn: negative name set of new tissue node (group);

• T: tissue ID of immediate sub-part of tissue identified by name sets and stage;

we can write a "query" in the form:

immediate_subPart_ns(17, ["embryo", "mesenchyme", "myocoele",

"paraxial mesenchyme", "somite", "trunk mesenchyme"],[], T), tissue(_, T, FN),

writeName(FN), nl, fail.

and obtain the following result:

("embryo", "mesenchyme", "trunk mesenchyme", "paraxial mesenchyme", "somite", "somite 05", "myocoele") 

("embryo", "mesenchyme", "trunk mesenchyme", "paraxial mesenchyme", "somite", "somite 06", "myocoele") 

...

("embryo", "mesenchyme", "trunk mesenchyme", "paraxial mesenchyme", "somite", "somite 30", "myocoele")

Similarly, using predicate immediate_superPart_ns(), we obtain:

("embryo", "mesenchyme", "trunk mesenchyme", "paraxial mesenchyme", "somite")

immediate_superPart_ns() is analogous, and its Prolog implementation very similar, to immediate_subPart_ns(). Details are, therefore, omitted.

The correctness of these results was confirmed by one of the biologists who created EMAP. Other, similar tests, worked equally well. A constraint put on all of these cases, however, is that the name set of the new group tissue must only contain names that are already used in the existing hierarchy.

This raises the question of how to deal with the introduction of new component names. For example, the addition of a group (embryo, head) cannot automatically be carried out, since the existing hierarchy does not use head in its name sets. For the integration to work, it is first necessary to add head to the appropriate name sets in the existing hierarchy. This can be done at the highest appropriate levels, since sub-parts inherit all name set elements from their super-parts, and may therefore not require as much effort as one initially expects.

For the head example, however, we did identify two additional problems which are likely to be typical in this context. Firstly, some agreement needs to be reached as to what in fact is considered to be part of the newly introduced tissue. In our example: how much of the neck is anatomically considered to be part of the head? The second problem deals with the fact that an existing tissue may need to be divided further in order to obtain the appropriate subparts for the newly introduced tissue. For example, the carotid artery runs from the head into the body of the mouse embryo, i.e. only a part of carotid artery is actually part of the head. Hence, the carotid artery needed to be divided into two subparts, one for the head section of it, one for the rest. In our name set approach, the former contains head in its positive name set, while the latter contains head in its negative name set. Of course, only the head section part becomes part of the head. Neither of these two problems presents any direct consequences for our approach.

When merging ontologies of different granularity, the same principle as before applies: shared component names must be used in a consistent manner. Assuming ontology *O*_1 _includes midbrain as one of the parts of the brain, but no further detail, and *O*_2 _is a brain anatomy ontology that divides the midbrain into cerebral aqueduct, floor plate, lateral wall, etc., then we would find {brain, central nervous system, embryo, midbrain, mouse, nervous system, organ system} as the positive name set for midbrain in *O*_1_, and {brain, cerebral aqueduct, midbrain} as the positive name set in *O*_2_, resulting in {brain, central nervous system, cerebral aqueduct, embryo, midbrain, mouse, nervous system, organ system} – the union of these previous two name sets – as the representation of midbrain in the merged ontology. The meaning of the component names in the intersection of the two original names sets, {brain, midbrain} must have been used in a consistent manner for the merger to work, though many of the component names will differ across the ontologies, because of the different levels of granularity, e.g. the terms nervous system and organ system are unlikely to be found in the brain specific ontology. (We omitted the negative name sets from this discussion, but the implications are essentially the same as for the positive name sets.)

Taking a closer look at these "basic tissue terms", called *component names *thus far, shows that some of them have additional structural complexity and if one wishes to take advantage of the semantics of these complexities, the proposed name set representation would need to be extended. For example, at Theiler stage 18 the tissue (embryo, branchial arch, 1st arch, mandibular component, mesenchyme) has two subparts, called (..., mesenchyme derived from head mesoderm) and (..., mesenchyme derived from neural crest). The naming, hence, reflects lineage relationships between tissues, and the identity of a tissue is partially established by that relationship. Although extensions to the name set representation could be developed to allow the inclusion and subsequent reasoning over such information, it would lead to a semantic overloading of the name sets and for simplicity are, therefore, not considered further – the (component) name is treated as an atomic string describing a tissue, while the lineage relationship is modelled externally to the name sets.

Theoretically, merging two part-of hierarchies can be accomplished by systematically (top-down) adding each tissue from one hierarchy into another, i.e. conceptually the problem can be reduced to iteratively adding "group nodes" as discussed above.

The approach discussed in this paper will not work where there has been no agreement on the basic component terms, and as such is different from already existing work on merging autonomous ontologies. This raises two questions: what is the basis on which these terms should be agreed and what benefits are to be obtained from the proposed solution if such agreement has to be reached before these partonomic hierarchies can be merged. With respect to the first question, if a basic term, for example skin, exists, then it must be possible to dissect the mouse to a level that separates all the corresponding tissue from the rest of the mouse tissues, e.g. separate all skin tissue from the rest of the mouse. Other examples of basic terms are, therefore, head, skeleton, limb and forelimb. At this point scientists are then free to use combinations of these terms (for the positive and negative name sets) to describe the anatomical concepts they are interested in, e.g. {head, skin} to refer to the skin of the head. The different anatomy hierarchies created by different scientists can then be automatically merged using the approach proposed in this paper. Hence, to answer the second question from above, the benefit of our solution lies in the removal of the need for multiple scientists to agree on a single anatomy partonomy where all tissue concepts are defined and their *part-of *relationships specified. Instead, a much more flexible solution is offered without having to sacrifice the interoperability across multiple data sets annotated with these anatomical concepts.

Essentially, the solution is based on the transitivity property of the structural part-of relationship. As such, one could imagine implementations other than the one based on name sets to achieve the same result. The basic idea, however, would be the same. Using the name set concept makes the solution more directly accessible to biologists, who are more familiar with naming anatomical concepts than using computer generated IDs. We believe that the same approach may be applicable in other ontology areas, which have similarly transitive relationships, but since we have not tested this idea, we shall not elaborate on it in this paper.

Also, the work described here only deals with the integration of hierarchies that are based on the same type of *part-of *relationships. Some preliminary studies suggest that where there are different types and these types are organised in an is-a hierarchy, the proposed integration mechanism will still work at the level of the common part-of type. For example, let H1 be a part-of hierarchy based on part-of-type-1, and let H2 be a part-of hierarchy based on part-of-type-2. If both, part-of-type-1 and part-of-type-2, are specialised versions of the more general part-of-type-0, i.e. part-of-type-1 is-a part-of-type-0 and part-of-type-2 is-a part-of-type-0, then we can use the proposed approach to integrate H1 and H2. The integrated hierarchy, however, would only support part-of-type-0 semantics. Our work in this area is still in its early phase and beyond the scope of this paper. Further details will be reported elsewhere.

The work presented in this paper has focused on the issue of integrating different partonomic hierarchies in one species, mouse. We note that a similar approach may be useful when trying to integrate partonomic hierarchies across different organisms. This is subject of current research work, however, and will be reported on separately.

## Conclusions

Anatomy ontologies play an important role in bio-medical informatics. One of the key relationships modelled in such ontologies is that of *part-of*. For any given organism, however, there is more than one way to divide it into parts and subparts, thus leading to more than one valid partonomic hierarchy. To be able to interoperate between bioinformatics resources that make use of these anatomy ontologies, the corresponding hierarchies must be reconciled in some way. The paper addresses the problem that unique identifying names for tissues often reflect the partonomic hierarchies in which they are used. Although these names are in fact ordered sets (the order implying a particular hierarchy) of "component names", the order in these sets is not necessary to uniquely identify any tissue. Also, the sets of components in names can be used to derive all part-of relationships in the hierarchy. Based on these observations, we have developed a name set representation which facilitates integration of different partonomic hierarchies. Although this does not eliminate the requirement to agree on a set of suitable basic tissue terms and their meaning, it does remove the need to standardise the partonomic hierarchies. The proposed approach has been tested for the anatomy ontology of the Edinburgh Mouse Atlas. A Prolog prototype was implemented for evaluation purposes.

## Note

^1^*T*_*j *_is a *direct subpart *of *T*_*i*_, if *T*_*j *_is part of *T*_*i *_and there is no other tissue *T*_*k *_such that *T*_*j *_is part of *T*_*k *_and *T*_*k *_is part of *T*_*i*_. If such a tissue *T*_*k *_exists, *T*_*j *_is an *indirect subpart *of *T*_*i*_.

## Authors' contributions

AB developed the name set representation, implemented the prototype and carried out parts of the evaluation. YY provided input with respect to the current implementation of EMAP and EMAGE in relation to the proposed name set representation. DD carried out part of the evaluation process. RB contributed to the development of the name set representation. DD and RB are overall project leaders of EMAP and EMAGE. All authors have contributed to the writing and/or revision of the paper.
